# Synthetic developmental biology: molecular tools to re-design plant shoots and roots

**DOI:** 10.1093/jxb/erad169

**Published:** 2023-05-08

**Authors:** Elif Gediz Kocaoglan, Dhanya Radhakrishnan, Naomi Nakayama

**Affiliations:** Department of Bioengineering, Centre for Synthetic Biology, Imperial College London, London SW7 2AZ, UK; Department of Bioengineering, Centre for Synthetic Biology, Imperial College London, London SW7 2AZ, UK; Department of Bioengineering, Centre for Synthetic Biology, Imperial College London, London SW7 2AZ, UK; Centre for Research in Agricultural Genomics (CRAG), Spain

**Keywords:** Growth and development, Phytobricks, plant synthetic biology, precision breeding, synthetic developmental biology, synthetic morphology

## Abstract

Plant morphology and anatomy strongly influence agricultural yield. Crop domestication has strived for desirable growth and developmental traits, such as larger and more fruits and semi-dwarf architecture. Genetic engineering has accelerated rational, purpose-driven engineering of plant development, but it can be unpredictable. Developmental pathways are complex and riddled with environmental and hormonal inputs, as well as feedback and feedforward interactions, which occur at specific times and places in a growing multicellular organism. Rational modification of plant development would probably benefit from precision engineering based on synthetic biology approaches. This review outlines recently developed synthetic biology technologies for plant systems and highlights their potential for engineering plant growth and development. Streamlined and high-capacity genetic construction methods (Golden Gate DNA Assembly frameworks and toolkits) allow fast and variation-series cloning of multigene transgene constructs. This, together with a suite of gene regulation tools (e.g. cell type-specific promoters, logic gates, and multiplex regulation systems), is starting to enable developmental pathway engineering with predictable outcomes in model plant and crop species.

## Teaser

As synthetic biology makes complex molecular engineering faster and more accessible for plant systems, we can start to re-design plant growth and development for climate-resilient agriculture.

## Introduction

Since the dawn of agriculture, we humans have sought to alter plant shape and architecture. Agricultural yield potential is tightly linked to plant growth and development. More fruits or grains would grow on a plant with more branches (Guo *et al.*, 2020; Zheng *et al.*, 2020). Harvest would increase if we could grow larger crops (Si *et al.*, 2016). Extensive root growth is likely to help with better soil anchorage, as well as nutrient and water absorption (Uga *et al.*, 2013; Li *et al.*, 2018). A major breakthrough in the original Green Revolution was the development of semi-dwarf varieties of cereal crops, which increased yield by reducing the damage due to structural failure (Khush, 2001). When shoots fall to the ground, excessive moisture can impair fruit development, while harvesting becomes challenging (Shah *et al.*, 2017). As the world population continues to rise rapidly, and with the imminent threats of climate change, we are in dire need of new technologies to achieve sustainable agricultural improvement. Reprogramming plant growth and development is key to the next Green Revolution, just like the first time.

The development of plant tissues or organs is wired through a complex molecular network involving parallel and converging pathways (Li *et al.*, 2004; Iwase *et al.*, 2011; Casanova-Sáez *et al.*, 2021), interplays with hormonal regulation (Durbak *et al.*, 2012), and feedback/feedforward loops (Benjamins and Scheres, 2008; Weijers and Wagner, 2016). Developmental processes progress with high spatial and temporal specificity, with different stages expressing distinct sets of genes (Koltunow *et al.*, 1990; Schmid *et al.*, 2005; Sparks *et al.*, 2013; Palovaara *et al.*, 2017). Furthermore, plant development adapts to the growth conditions, and thus the underlying molecular mechanisms have different versions depending on the environmental input (Shao *et al.*, 2007). Genetic interventions towards desirable traits have occurred throughout the history of crop domestication via breeding efforts and, in the past three decades, also with genetic engineering. However, precision engineering of pre-defined morphology and architecture remains a challenge, presumably because of the complexity of the pathways requiring tight spatiotemporal control and a high degree of robustness.

Synthetic biology refers to the rational engineering of gene regulatory networks to generate predictable outcomes in a biological host (chassis). It is also called ‘engineering biology’ for its application of engineering principles, such as standardization and design-led, iterative cycles of trials to bring the outcome closer to the purpose. Over the past two decades of synthetic biology, much effort has been made to create engineering resources for the precision genetic engineering of molecular pathways complicated enough to act like a circuit (Brophy and Voigt, 2014). In other words, synthetic biology opens new doors to a whole new level of genetic modification of biological development. Synthetic developmental biology is a frontier at the interface between synthetic biology and developmental biology in animal systems (Ebrahimkhani and Ebisuya, 2019; Zarkesh *et al.*, 2022). The time is also opportune for synthetic biological engineering of plant development.

In this review, we first provide an overview of synthetic biology tools that are likely to be useful for developmental reprogramming of plants. Then three case studies follow to illustrate the power of synthetic biology tools and strategies in rational modification of plant morphology. We conclude by highlighting future challenges and perspectives.

## DNA assembly frameworks and toolkits

In engineering, standardized, modular, and well-characterized building parts are used to construct a device. Similarly, in biology, how individual DNA parts function needs to be known in order to design or re-design biological systems rationally. With this goal, synthetic biology promoted standardization and characterization of DNA parts adhering to the BioBricks standard of DNA part construction (Knight, 2003). BioBricks are basic DNA parts with pre-defined flanking prefix and suffix sequences. These prefix and suffix sequences encode restriction enzyme recognition sequences, allowing users to create compatible sticky ends that self-assemble in specific combinations. For an extensive review of molecular cloning, readers are referred to Casini *et al.* (2015). Multiple BioBricks can be combined quickly and semi-scarlessly with Golden Gate technology (Engler *et al.*, 2008). Golden Gate frameworks use Type IIS restriction enzymes which cut DNA downstream of their recognition sequence; this allows users to design their 4 bp overhangs and assign an order to how the DNA parts will be assembled. Unique 4 bp overhang sequences were given to different types of DNA parts with the Phytobrick standard, facilitating a community-wide part exchange (Patron *et al.*, 2015). Although the plant community initiated the Phytobrick standard, the overhang sequences comply with the commonly used selections for other eukaryotic organisms (Cai *et al.*, 2020a). Phytobrick has been adopted widely in non-plant communities (e.g. iGEM) as a universal overhang format (https://2016.igem.org/Resources/Plant_Synthetic_Biology/PhytoBricks).

**Fig. 1.**
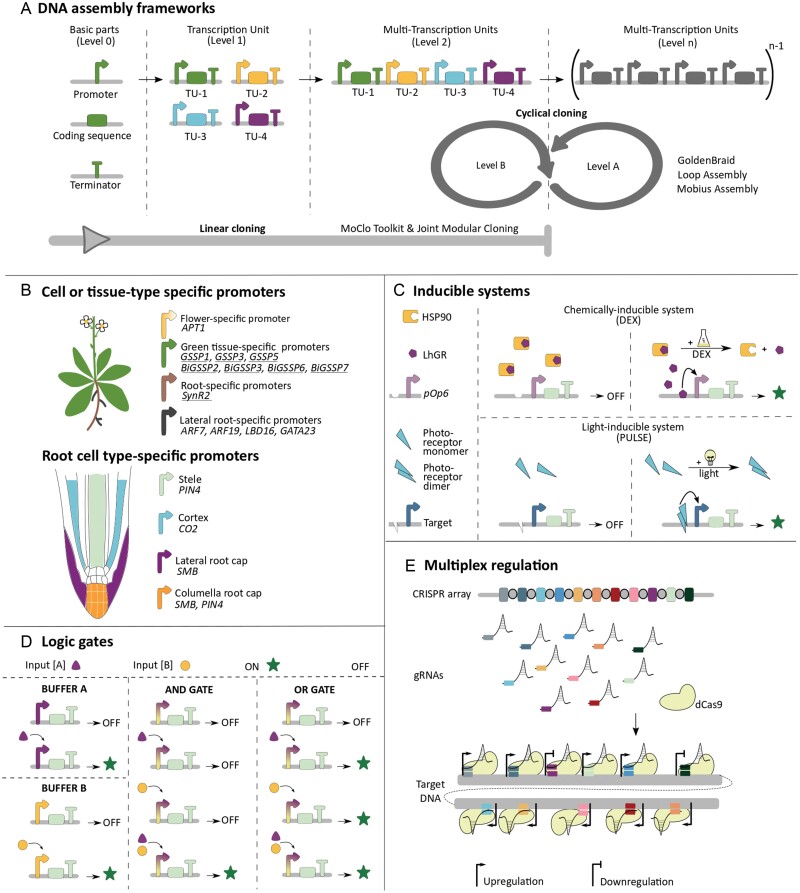
Major types of plant synthetic biology tools for developmental reprogramming. (A) DNA assembly frameworks. Basic parts form Level 0, and a transcription unit (TU) can be formed by combining a promoter, coding sequence, and terminator at Level 1. Multiple TUs can be combined at Level 2. Linear cloning systems (e.g. MoClo, Weber *et al.*, 2011 and Joint Modular Cloning, Chamness *et al.*, 2023) stop at Level 2. Cloning toolkits that follow cyclical cloning (e.g. GoldenBraid, Sarrion-Perdigones *et al.*, 2011, Loop, Pollak *et al.*, 2020, and Mobius Assemblies, Andreou and Nakayama, 2018; Andreou *et al.*, 2021, Preprint) can switch back and forth between Level A and B, allowing infinite expansion. Mobius follows hierarchical assembly until Level 2, then joins cyclical cloning. (B) Cell- or tissue type-specific promoters. Schematics of the non-constitutive promoters mentioned in this review. Rationally designed synthetic promoters are underlined. (C) Inducible systems. The left panel shows a chemically inducible system based on dexamethasone (DEX). Without DEX, LhGR is bound to HSP90 away from the nucleus. When DEX is added, LhGR is released and can bind to its binding site *pOp6*, inducing gene expression (Samalova *et al.*, 2005). The right panel shows a light-inducible system, the blue-light module from PULSE. Without blue light, the photoreceptor is a monomer and cannot bind to its target. When present, the photoreceptor forms a dimer and binds to its target, driving gene expression. In the PULSE system, the blue-light module is later fused to a repressor domain for the gene repression (Ochoa-Fernandez *et al.*, 2020). (D) Logic gates. The AND gate based on two inputs [A] and [B] is at its ‘ON’ state when both [A] and [B] are present. The OR gate is at its ‘ON’ state when [A] or [B] is present, and ‘OFF’ only when both inputs are absent. (E) Multiplex regulation. Multiple gRNAs can be expressed in a single CRISPR array and, with the presence of dCas9-i systems, multiple genes can be up- or down-regulated simultaneously. The diagram shows nine up-regulations and two down-regulations reported in *S. cerevisiae* (Shaw *et al.*, 2022).

Over the years, multiple variants of Golden Gate-based cloning toolkits have been developed for plant systems, summarized in Table 1. One of the earliest toolkits is MoClo Assembly, and it implemented a linear strategy to Golden Gate, where there is a hierarchy of Levels 0, 1, and 2 (Weber *et al.*, 2011). In MoClo, basic DNA parts (Phytobricks) comprise Level 0. When basic DNA parts such as a promoter, coding sequence, and terminator are combined, it forms a functional transcription unit (TU) at Level 1. Multiple TUs can be combined at Level 2 to create a multigene construct (Fig. 1A). MoClo has intermediate modules that provide end-linkers for expanding cloning capacity beyond Level 2. DNA assembly toolkits for plant gene expression control, such as the Plant MoClo toolkit, use *Escherichia coli* as a propagation host. *Escherichia coli* grows faster, and plasmid purification yields are higher than other bacterial species, such as *Agrobacterium tumefaciens*—the most commonly used bacterium for gene transfer into plant cells. *A. tumefaciens* can insert the DNA fragment between the left border (LB) and right border (RB) T-DNA insertion sequences into the host plant genomes. The vectors in plant DNA assembly toolkits accept TUs cloned between LB and RB sequences. The natural hosts of *A. tumefaciens* are dicots rather than monocots. Thus, the plasmids based on *A. tumefaciens* transformation work well for dicots but not for monocots (Sood *et al.*, 2011). Recently, the Joint Modular Cloning (JMC) Toolkit has been developed, which follows the same linear strategy as MoClo, but with a higher capacity (Chamness *et al.*, 2023). The JMC toolkit uses pMIN vectors which can be employed to transform both *Nicotiana benthamiana* (dicot) and *Setaria viridis* (monocot).

**Table 1.** Golden Gate-based cloning toolkits for plant systems

Toolkit	Vector	Strategy	Capacity	Part types	Highlights	Reference
**MoClo Assembly**	pGreen+pSoup	Linear	7 transcriptional units (TUs), can be increased by auxiliary modules	Natural and synthetic promoters, integrase-based toolbox, recombinase-based logic gates	Widely used but has 53 acceptor vectors to choose from, which makes cloning complicated	Weber *et al.* (2011)
**Joint Modular Cloning (JMC)**	pMIN	Linear	7 TUs, can be increased by expansion modules	Monocot and dicot promoters, terminators, enhancers	Shown to work in both monocots and dicots, uses an isoschizomer of *Aar*I (*Paq*CI) and uses expansion linkers to simplify combinatorial DNA assembly	Chamness *et al*, (2023)
**GoldenBraid**	pGreenII+pSoup or pCambia	Cyclical	Unlimited	Synthetic promoters	Initial assembly was pairwise which increases the number of cloning steps to achieve the final construct, fixed in GB2.0 and GB3.0	Sarrion-Perdigones *et al.* (2011)
**Loop Assembly**	pGreenII+pSoup pCambia	Cyclical	Unlimited	*Marchantia polymorpha* parts	Open MTA licence for unrestricted use but pCambia vectors have large backbone (~6.5 kb) which might cause stability issues	Pollak *et al.* (2020)
**Mobius Assembly for Plant Systems (MAPS)**	pMAPS	Cyclical	Unlimited	Small promoters, terminators, reporter genes	MethylAble for combinatorial DNA assembly, use of *AarI* as Type IIS restriction enzyme which reduces domestication	Andreou and Nakayama (2018); Andreou *et al.* (2021, Preprint)

Different from the linear cloning strategy of MoClo and JMC, GoldenBraid and Loop Assembly operate in a cyclical fashion, switching back and forth between two cloning levels (Sarrion-Perdigones *et al.*, 2011; Pollak *et al.*, 2020). GoldenBraid and Loop Assembly have two types of vectors: one for even levels (Level 0, 2, 4, … ) and one for odd levels (Level 1, 3, 5, … ). Because they can switch back and forth between the two levels, they have unlimited cloning capacity (Fig. 1A). Unlimited cloning capacity is a common feature of cyclic cloning systems. However, cloning capacity is often limited by the metabolic burden imposed by plasmid size and stability (Rouches *et al.*, 2022). Plant multigene constructs tend to be extensive (>20 kb) and, together with a plasmid having a large backbone, such as pCambia (~6.5 kb) used by GoldenBraid and Loop, cloning capacity could be compromised. Alternatively, these toolkits use smaller pGreen-type vectors (~5 kb), but they require an additional plasmid pSoup (~9.2 kb) to replicate in *A. tumefaciens* (Sarrion-Perdigones *et al.*, 2011; Pollak *et al.*, 2020). An advantage of Loop Assembly is that vectors and parts in the toolkit are under OpenMTA, which enables unrestricted use.

Mobius Assembly is another framework for plant systems that combines linear and cyclical strategies. Mobius Assembly has only one vector (mUAV) to store basic parts and follows a linear strategy up to Level 2. For higher level constructs, users can switch back and forth between Level 1 and Level 2 vectors (cyclical cloning) (Andreou and Nakayama, 2018). Mobius Assembly additionally uses a unique Type 2SII restriction enzyme, *Aar*I, which has a 7 bp recognition sequence. The use of *Aar*I reduces domestication (removal of internal restriction enzyme sites) by 58.3% compared with systems using three 6 bp cutters, such as GoldenBraid and Loop Assembly (Andreou *et al.*, 2021, Preprint). Mobius Assembly also offers the MethylAble feature; Phytobricks are flanked by two divergent *Bsa*I recognition sites that are prone to methylation and protected from digestion, while the convergent sites are not. This allows Level 0 parts to be combined with pre-made Level 1 constructs or fused with a Level 2 TU, simplifying combinatorial DNA library construction.

Development of Golden Gate-based cloning toolkits for plants enabled simple and fast multigene assembly. Each toolkit possesses distinctive features, and the decision to use a specific toolkit depends on the planned molecular construction. JMC would be preferred when working with monocots and dicots. Mobius may be the most efficient in building a series of constructs that differ in one part, especially for part characterization, thanks to the MethylAble feature.

## Tools for gene regulation

Characterization of DNA parts typically involves quantitation of reporter gene expression, where different DNA parts are combined to drive the output. Well-characterized DNA parts enable users to tune gene expression levels by changing the promoters, terminators, and other regulatory sequences (e.g. enhancers) predictably (Schaumberg *et al.*, 2016). Thorough characterization of such basic parts is crucial because gene expression strength varies depending on the nature of the protein and application. Additionally, it is becoming increasingly clear that the same construct can lead to different levels of activities in different chassis types (Wilmink *et al.*, 1995; Chamness *et al.*, 2023). The desired range for the selected organism can be achieved by changing the combinations of the basic and regulatory parts, and having well-characterized parts allows for a more predictive construct design.

### Generic promoter and terminator parts

DNA assembly toolkits included in Table 1 (MoClo, JMC, GoldenBraid, Loop Assembly, and Mobius Assembly) adhere to the Phytobrick design standard; thus, they are compatible. Collectively, these toolkits offer 41 constitutive promoters and 41 terminators that have been characterized in *A. thaliana* and *N. benthamiana,* which are both dicot plants (Sarrion-Perdigones *et al.*, 2011; Weber *et al.*, 2011; Andreou and Nakayama, 2018; Pollak *et al.*, 2020; Andreou *et al.*, 2021, Preprint; Chamness *et al.*, 2023). JMC contains six monocot promoters characterized in *S. viridis*. Regulatory parts can behave distinctly in monocots compared with dicots; for example, the most widely used dicot promoters, 35S and NOS, showed weak expression in *S. viridis.* It has been known that the 35S promoter has lower activity in monocot species compared with dicots. The JMC toolkit further highlights that the expression strength of DNA parts can be species dependent, and partial characterization should be conducted in each species of interest without the assumption of orthogonality (Wilmink *et al.*, 1995; Chamness *et al.*, 2023). Orthogonality is the ability of DNA parts to operate in a defined way, independent of the contexts and backgrounds; this is one of the most sought-after traits in synthetic biology. Furthermore, not only promoters control gene expression, but terminators are also becoming recognized for their roles in gene regulation. The part characterization for Mobius Assembly for Plant Systems (MAPS) showed synergistic interactions between promoters and terminators. Promoter strength can change 5-fold when combined with 14 different terminators tested (Andreou *et al.*, 2021, Preprint).

In addition to directly utilizing natural promoter or terminator sequences from plants or plant pathogens, one can rationally design synthetic parts. Rationally creating synthetic parts can facilitate orthogonality, minimizing the presence of unknown functional sequences and the size of the plasmid. A synthetic promoter typically has a core promoter region with a TATA-box for transcription initiation and has *cis*-acting elements (the binding sites of transcriptional regulators, i.e. *trans*-elements). TATA-box location, GC content, and *cis*-acting elements can be changed to build synthetic promoters with predicted strength (Cai *et al.*, 2020b; Jores *et al.*, 2021; Moreno-Giménez *et al.*, 2022). There are 59 synthetic promoters characterized for use in plants built with DNA assembly frameworks mentioned in this work. Synthetic terminators for plants are yet to be developed and characterized, but they are available for yeast *Saccharomyces cerevisiae* (Curran *et al.*, 2015).

The two most used chassis for part characterization are the transient expression system in leaf (commonly in *N. benthamiana*) and protoplasts. For leaf infiltration, a construct harbouring the part to be characterized is inserted into *A. tumefaciens* (Li, 2011). *A. tumefaciens* is used to infiltrate the leaves using a needleless syringe. At 4–5 d after infiltration, leaves are either imaged for a visible reporter activity, or the reporter molecules are extracted for spectrophotometer quantification. The second method, protoplast transient expression, requires protoplast isolation (Yoo *et al.*, 2007). Plant leaves are collected, and cell walls are enzymatically digested to generate cells without walls (protoplasts). Then the construct is transformed into the protoplasts by either electroporation or polyethylene glycol (PEG) treatment. At 12–24 h after transformation, the protoplasts produce enough reporter molecules for quantification. The protoplast transient expression can be used for both monocots and dicots; however, protoplast isolation and transformation are challenging due to the fragile nature of protoplasts. On the other hand, leaf infiltration so far is limited to the dicot chassis and may not be suitable for characterizing monocot parts.

Both characterization platforms are prone to experiment to experiment variations. In the leaf infiltration system, *A. tumefaciens* distributes unevenly within a leaf, and plasmid numbers vary from cell to cell (Bashandy *et al.*, 2015). Protoplast preparation stresses the cells greatly, and the transformation efficiency and transgene expression can be inconsistent between batches. To overcome such experimental variations, the standard part characterization protocol recommends a dual-reporter system (Schaumberg *et al.*, 2016). Reporter systems are usually luciferase based [(e.g. nanoluciferase (Nluc) and firefly luciferase (Fluc)] or fluorescent protein based [e.g. green fluorescent protein (GFP) and red fluorescent protein (RFP)]. The first reporter acts as the normalizing construct (Fluc), which is the same for each sample. The second reporter contains the part to be characterized (Nluc), which is expressed under the control of the regulatory part under characterization. The normalized strength of a promoter or terminator part can be determined by calculating the fluorescence ratios of the two reporters (Nluc/Fluc). Even though this method is mainly used to analyze constitutively expressing parts, the same strategy can be applied to characterizing a standard part within a specific cell or tissue context.

### Cell- or tissue-type-specific promoters

Developmental engineering requires spatiotemporal control over gene expression. Therefore, one of the essential tools for engineering plant development is cell- or tissue type-specific promoters. Organismal development is a temporally and spatially dynamic process rich in cell- and tissue type-specific gene activities. Constitutive expression of developmental genes throughout a multicellular organism can be deleterious, leading to pleiotropic or embryo-lethal phenotypes. It is advisable to confine the expression to specific cell types or tissues, such as the meristems [shoot apical meristem (SAM), root apical meristem (RAM), or cambium], roots, shoots, flowers, or leaves, and distinct cell types therein (Nalapalli *et al.*, 2021).

**Fig. 2.**
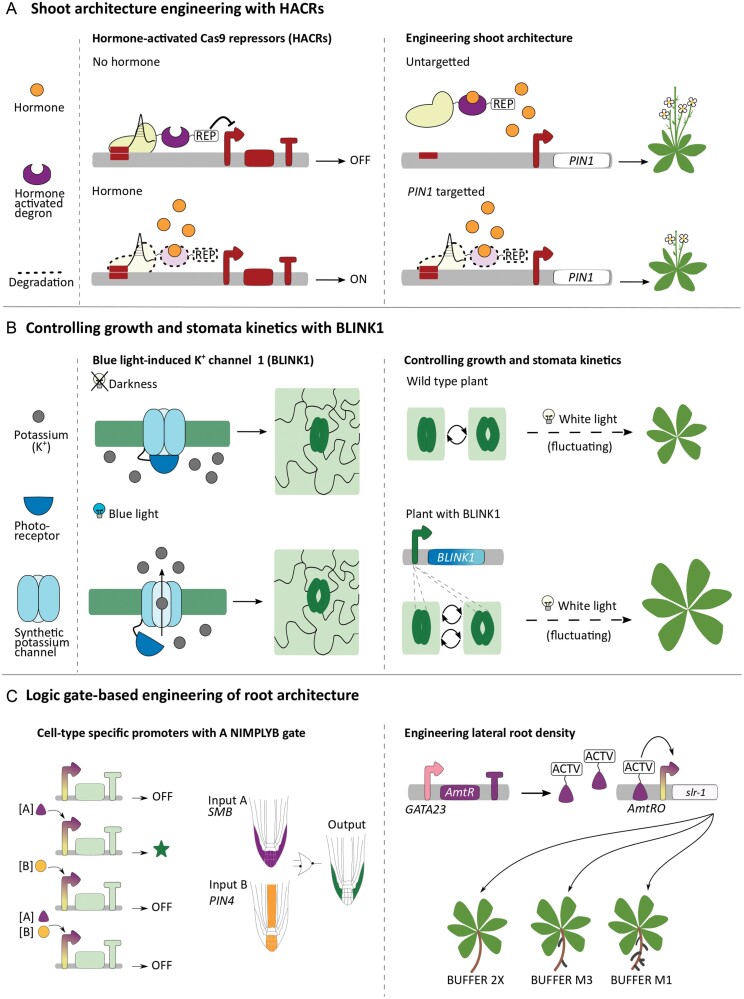
Case studies showcasing the engineering of plant development using synthetic biology. (A) Shoot architecture engineering via the hormone responses (Khakhar *et al.*, 2018). The gRNA targets hormone-activated Cas9 repressors (HACRs) to the promoter of interest, where the TPL represses gene expression. The auxin-induced degron enables ubiquitination of HACRs upon auxin accumulation, which leads to degradation of the HACR complex. When auxin accumulates, the target gene is activated. When HACRs were targeted to *PIN-FORMED 1* (*PIN1*), which mediates auxin movement across a tissue, they dampened the *PIN1*–auxin feedback loop, causing plants to be shorter and have less branching. (B) Growth control with a synthetic ion channel modulating stomatal opening (Papanatsiou *et al.*, 2019). BLINK1 is a synthetic potassium channel fused to a plant-derived photoreceptor. The photoreceptor inhibits the channel function in darkness or the absence of blue light. When the blue light is present, the photoreceptor changes its confirmation and triggers channel function. This induces potassium efflux out of the cell, hyperpolarizing the cell membrane and opening the stomata. When BLINK1 is expressed under a guard cell-specific promoter, the potassium channel function in stomata can be controlled with blue light illumination. BLINK1 improved stomata opening kinetics and carbon assimilation under fluctuating white light. As a result, BLINK1 plants have 2.2 times more biomass than wild-type plants. (C) Root architecture engineering with logic gates (Brophy *et al.*, 2022). Cell type-specific promoters were constructed using an ANIMPLYB gate to confine gene expression to one cell type. The ANIMPLYB gate activates the target gene when only [A] is present and represses it with both [A] and [B]. Designing the logic gate to respond to *SOMBRERO* (*SMB*) and *PIN4* expression could drive the reporter gene expression only in the lateral root cap. This system was used to tune the lateral root number via the expression of the mutant *IAA14* gene (*slr-1*), which blocks root branching. The synthetic promoter strength was adjusted to control the lateral root number; the copy number and sequence of the *AmtR* operator changed the promoter strength and the lateral root number.

There are well-characterized promoters that demarcate a specific cell or tissue type in the root, in which the radial cell layers have distinct identities (Fig. 2B). Marquès-Bueno *et al.* (2016) identified and characterized 28 different promoters varying in expression in different cell types in the *A. thaliana* root, such as the stele, cortex, and quiescent centre. Even though their expression can be limited primarily to a specific cell type in the root, these promoters typically have activities elsewhere; for example, *PIN4*, mainly expressed in columella root cap and stele, is also expressed in the leaves and flowers of mature plants, and is involved in embryogenesis (Fig. 1B) (Klepikova *et al.*, 2016). In addition to the cell types in the primary root, Guiziou *et al.* (2023) have developed an integrase-based MoClo toolbox to modulate gene expression during lateral root formation. They have used promoters of *AUXIN RESPONSE FACTOR 7* (*ARF7*), *AUXIN RESPONSE FACTOR 19* (*ARF19*), *LATERAL ORGAN BOUNDARIES DOMAIN 16* (*LBD16*), and *GATA TRANSCRIPTION FACTOR 23* (*GATA23*) transcription factor genes (Fig. 1B). Similarly, these transcription factor genes show expression in mature leaves and flowers, although weaker than *PIN4* (Klepikova *et al.*, 2016). The basal level expression might be negligible if a threshold gene expression is needed for a developmental switch (e.g. the *LEAFY* gene in reproductive development); if the presence or absence of a gene activity determines the shift, then such baseline expression could be detrimental (e.g. *TOPLESS* in embryogenesis) (Blazquez *et al.*, 1997; Long *et al.*, 2006).

One strategy to overcome the limitations of endogenous cell- or tissue type-specific promoters is to design synthetic promoters rationally. Rationally designed tissue-specific promoters are summarized and underlined in Fig. 1B. Light-responsive *cis*-acting elements are incorporated into the synthetic promoters to confer green tissue specificity, as shown with *GSSP1,3,5* promoters (R. Wang *et al.*, 2015). As the leaf, sheath, panicle, and stem are all green, chlorophyll-containing tissues, the transgene expression cannot be restricted to one type of tissue yet; however, expression strength can be varied between the green tissues with *BiGSSP2,3,6,7* (Bai *et al.*, 2020). Similarly, the *SynR2* synthetic promoter is built for root expression, but its expression cannot be limited to one cell type, and there is a basal level of expression in the leaf, stem, and seed (Mohan *et al.*, 2017). Developments in single-cell transcriptional profiling technologies and identification of new, *cis*-acting elements would facilitate rational designing of cell type-specific promoters in the future.

### Inducible systems

In addition to tissue type-specific promoters, inducible systems can provide spatiotemporal regulation. A typical inducible system will have an inducible promoter, which will drive the expression of the target gene in the presence of a trigger input, such as an inducer chemical, heat shock, or wounding.

Ethanol (Caddick *et al.*, 1998), β-oestradiol (Zuo *et al.*, 2000), and dexamethasone (DEX) (Samalova *et al.*, 2005) are the most used chemically inducible systems in plants. They have distinct mechanisms of gene activation. The ethanol-inducible system was isolated from the fungus *Aspergillus nidulans*, and AlcR protein binds to the *AlcA* promoter in the presence of ethanol (Caddick *et al.*, 1998). Originally developed >20 years ago, the ethanol-inducible system was recently shown to cause growth defects in plants (Randall, 2021). The β-oestradiol and DEX systems act through a chimeric receptor. The chimeric receptor for the β-oestradiol system is composed of the DNA-binding domain of a bacterial transcription factor, the VP16 activator domain, and the regulatory region of the human oestrogen receptor (Zuo *et al.*, 2000). In the presence of β-oestradiol, the chimeric receptor changes its conformation, moves to the nucleus, and binds to its target promoter. The DEX-inducible system works similarly. The transcription activator LhGR is retained in an inactive form by the HSP90 protein. When DEX is present, HSP90 releases LhGR, which moves from the cytosol to the nucleus, binds to its *cis*-element *pOp6*, and activates transcription of the target gene (Fig. 1C).

Chemically inducible systems are challenging to control due to diffusion through cell layers precisely, and they lack cell and tissue type specificity by default. However, chemically inducible systems can be combined with cell- or tissue type-specific promoters. Schürholz *et al.* (2018) created 19 driver lines, in which a tissue-specific promoter drives the expression of LhGR in a distinct cell type in the root, shoot, SAM, procambium, and xylem/phloem precursors. In the absence of the inducer, LhGR is retained in the cytoplasm and kept inactive. They express effectors under the control of the *pOp6* promoter. Only if DEX is applied to where the driver is present is the effector expression activated. A downside of this system is that crossing and generating F_1_ lines is necessary and, thus, time-consuming.

In addition to chemically inducible systems, optogenetics allows gene activation on light cues, combining tight spatiotemporal control and reversibility. Implementing optogenetic switches in plant systems has been challenging however, as plants naturally respond to a broad spectrum of light. In fact, most optogenetic light sensors originate from plant genes. The PULSE system has overcome this by using two engineered photoreceptors: the blue-light module repressing gene expression in the presence of blue light, and the red-light module activating gene expression only in the presence of monochromatic red light (Ochoa-Fernandez *et al.*, 2020). The blue-light module is based on the dimerization of photoreceptors. Without blue light, the photoreceptor is in its monomer form and cannot bind to its target domain. When blue light is present, the photoreceptor dimerizes and binds to its target (Fig. 1C). The red-light module is based on attaching a different photoreceptor to its DNA-binding domain when red light is present. When the blue-light module has a repressor domain while the red-light module has an activator domain, the blue-light module dominates and represses the target gene expression. Thus, the target gene stays inactive when plants grow under white light (or in darkness). The PULSE system, in combination with CRISPR/Cas9 [clustered, regularly interspaced, short, palindromic repeats)/CRISPR-associated protein 9], was demonstrated to control transient gene expression in *N. benthamiana* leaves and *A. thaliana* protoplasts.

An ideal inducible system for developmental engineering would be free of any basal-level activity when the inducer is absent while being able to induce gene expression on cue in a specific tissue or cell type. Optogenetic inducible systems outperform chemical systems in terms of leakiness and spatiotemporal specificity (de Mena *et al.*, 2018; Pérez *et al.*, 2022). Optogenetic switches tend to be binary (on/off), and the target tissue or cell types must be accessible to light (i.e. close to the surface). A binary mode might be preferable in cases where a low level of expression of a gene can trigger a developmental switch. If a dynamic range is needed, chemically inducible systems may be preferable. A dynamic range is useful in modifying dose-dependent pathways [e.g. plant hormones such as auxin control developmental switches throughout the plant life cycle, from embryogenesis to root and shoot patterning, through dynamically changing local accumulation (Moller and Weijers, 2009; Kieffer *et al.*, 2010)]. Another feature to consider when choosing an inducible system is reversibility. Some developmental genes need to be only transiently active (e.g. in regenerating Arabidopsis roots, *PLT2* needs to be expressed only over a specific time frame to allow shoot initiation from callus, but continuous expression inhibits shoot formation) (Kareem *et al.*, 2015).

### Logic gates

Logic gates are genetic regulatory networks that mimic electronic circuits; they perform logical calculations based on the presence or absence of certain inputs and give a binary output (on/off) (Boolean algebra). Inducible systems can be engineered to function as logic gates, which can be used to build genetic circuits. Recently logic gates for plant systems were developed (Brophy *et al.*, 2022; Lloyd *et al*., 2022). A simple logic gate is ON when its designated input is present and OFF when the input is absent. Logic gates can be built to perform more complicated calculations such as AND, OR (demonstrated in Fig. 1D), and NOR and IMPLY (‘if Input [A], Output [B]’). The functionality of these logic gates has been tested in transient assays with *A. thaliana* protoplasts or *N. benthamiana* leaves or stable transformants of *A. thaliana*.

Epigenetic regulation often underlies establishing and maintaining cell fates, as well as environmentally induced developmental reprogramming. Logic gates based on transient expression of inducers or target genes lack memory, as the gate will reverse its output when the inducer availability changes. Recombinase-based gene circuits provide an alternative to engineering cell fate inheritably and for lineage tracking. A recently developed recombinase-based logic gate works by adding a terminator between the promoter and coding sequence, thereby repressing activation. Recombination signal sequences flank this terminator and, when the recombinase is active, it removes the terminator and thus initiates the target gene expression (Lloyd *et al.*, 2022). This system uses AND/OR gates (Fig. 2D) with two recombinases: one is expressed under a tissue-specific promoter as Input [A] (the cortex-specific *CO2* promoter, Fig. 2B), and the second one is expressed under a chemically inducible promoter as Input [B] (DEX-inducible *pOp6* promoter, Fig. 1C). Therefore, gene expression will only occur in the cortex cells supplied with DEX. This system was built by MoClo assembly and shown to work in *A. thaliana* roots with fluorescent proteins as the readout.

### Multiplex regulation

In addition to modulating the expression of one gene at a specific time and space, plant development often requires simultaneous regulation of multiple genes. Deactivated CRISPR (dCas)-based multiplex gene regulation systems have been developed and proven effective for plant systems. Unlike nuclease-active Cas9 used for genome editing, dCas retains the ability to bind its guide RNA (gRNA) and target DNA sequence, but does not cleave the target DNA. When dCas is fused to an activation domain (e.g. VP16, ERF2, or EDLL), dCas9 brings the transcriptional machinery to its target promoter and up-regulates gene expression. Similarly, gene repression can be achieved by fusing with a repression domain (e.g. BRD, TPL, or SRDX). Alternatively, genes can be repressed by simply using the dCas–gRNA pair alone because dCas9–gRNA makes the target promoter inaccessible to the host transcriptional machinery (Moradpour and Abdulah, 2020). The recently developed CRISPR–Act3.0 system could simultaneously activate six genes in rice (Pan *et al.*, 2021). Multiplex activation was demonstrated in *A. thaliana* by targeting two developmental regulator genes *FLOWERING LOCUS T* (*FT*) and *CL1*. Three gRNAs repressing the exogenous NOS promoter have been reported for multiplex repression using dCas12a fused to the BRD repression domain (GoldenBraid 4.0) (Vazquez-Vilar *et al.*, 2021).

In plant systems, combinatorial activation and repression (mixed-mode multiplex) has been limited to targeting one gene for activation and one for repression (Lowder *et al.*, 2017). However, in the yeast *S. cerevisiae*, recently 11 genes could be targeted, up-regulating nine and down-regulating two simultaneously (Fig. 2E). This system uses 24 gRNAs on a single array, which was assembled by Golden Gate cloning (Shaw *et al.*, 2022). Multiplex repression seems more challenging in plant systems than in *S. cerevisiae*.

## Case studies

In the past 5 years, we are seeing more rational engineering of plant growth and development. Below we spotlight three showcase examples where synthetic biology technologies, especially the types of molecular tools mentioned above, were effectively used to modify plant morphology.

### Shoot architecture engineering with hormone-activated Cas9 repressors (HACRs)

One of the first examples of synthetic biology applied to modulate plant development was HACRs (Khakhar *et al.*, 2018). HACRs combine Cas9, an auxin-induced degron domain, and the TPL repressor domain to regulate gene expression. Cas9 targets HACRs to the promoter of interest, where the TPL domain represses gene expression. The auxin-induced degron domain enables ubiquitination of HACRs upon auxin accumulation, leading to protein degradation. Therefore, where auxin accumulates, TPL is degraded, and the target gene expression is relieved from repression (Fig. 2A). As the proof-of-concept, HACRs were used to regulate the *PIN-FORMED 1* (*PIN1*) promoter, which mediates the polar transport of auxin within the cell and accumulation dynamics at the tissue scale. In line with the prediction of computational shoot architecture models (Prusinkiewicz *et al.*, 2009; Refahi *et al.*, 2016), Arabidopsis plants with *PIN1*-targeted HACRs displayed a reduction in the strength of the auxin–PIN1 feedback loop, generating a reduced number of branches. By lowering the transcriptional feedback strength, the irregularity in the phyllotactic pattern that arises stochastically from the anomalous auxin was reduced.

HACRs have modular hormone-induced degron domains, which can be swapped to respond to jasmonate and gibberellin, or alternatively to auxin. The modular and conserved nature of HACRs makes them useful for rewiring connections between hormone circuitry and developmental regulators.

### Growth modulation via stomatal opening with synthetic ion channels

Another example of synthetic proteins developed to alter plant morphology is the blue light-inducible potassium (K^+^) channel 1 (BLINK1), which is based on light-inducible control of ion channels (Papanatsiou *et al.*, 2019). The light inducibility is built by fusing a plant photoreceptor module (LOV domain) to a viral potassium channel. In the darkness, the photoreceptor module binds to the catalytic domain of the potassium channel and blocks potassium export. When illuminated with blue light (or white light, which contains blue-range wavelengths), the photoreceptor module changes its conformation, frees the catalytic domain, and triggers potassium transport out of the cell (Fig. 2B). As optogenetic systems are reversible, when illumination is off, the photoreceptor module recovers and switches back to its initial confirmation, inhibiting the export function. When BLINK1 was expressed under a guard cell-specific promoter (*pMYB60*), its light-responsive function was restricted to stomata, while the potassium channel activity in the rest of the plants functioned normally. As the stomatal aperture can be controlled by the polarization of the guard cell membrane, BLINK1 can control stomatal opening kinetics. Under fluctuating white light (mimicking light fluctuations in nature), the stomatal opening of the BLINK1 plants was faster, and their biomass was 2.2 times more than that of wild-type plants (Fig. 2B). The increase in their biomass was attributed to more efficient carbon assimilation without compromising water use (Papanatsiou *et al.*, 2019).

This study presents a novel strategy to enhance carbon assimilation and plant growth under changing environmental conditions. Implementation of BLINK1 to crop species would probably improve agricultural production. The BLINK1 system and similar light-induced synthetic ion channels for other signalling molecules, such as calcium ions, could provide new strategies for engineering plant growth and development, as well as sensing and responding to environmental stressors.

### Logic gate-based engineering of root architecture

Another example that integrated multiple synthetic biology tools to engineer plant development is the logic gates study by Brophy *et al.* (2022). They combined synthetic activators, repressors, and their target promoters to develop 10 synthetic transcription factors. After characterizing these synthetic transcription factors, the best performing AmtR-VP16 transcription factor (which contains the VP16 transcriptional activator domain) is used to build an ANIMPLYB gate (Fig. 2C). In an ANIMPLYB gate, gene expression will be turned ON when only Input [A] is present and will be OFF when both Inputs [A] and [B] are present. Using *SOMBRERO* (*SMB;* specific to the lateral and columella root cap) expression as Input [A] and *PIN4* (specific to the lateral and columella rot cap) expression as Input [B], they were able to confine GFP expression to only the lateral root cap (Figs 1B, 2C). Using logic gates in this way presented a novel strategy for developing tissue-specific promoters.

As a pilot study of logic gate-enabled plant developmental engineering, these logic gates were used to engineer root architecture. The *GATA23* promoter is active predominantly in the lateral root stem cells (Fig. 1B). The *GATA23* promoter was designed to drive the expression of the Amtr-VP16 activator, which is the input to the BUFFER gate. AmtR-VP16 then switches on the expression of a mutant *IAA14* gene (*slr-1*), which blocks root branching. The strength of the synthetic promoter was tuned by changing the copy number of the *AmtR* operator (BUFFER 2X gate) and mutating nucleotide sequences of the AmtR-binding site (BUFFER M3 and BUFFER M1) in synthetic promoters. With this construct, Arabidopsis plants with varying levels of lateral root density could be generated.

This study systematically applied engineering principles to rational modification of plant development through multiple rounds of design–build–test–learn cycles. It also addressed how fine-tuning of gene regulatory tools is required, especially in the different species and when shifting between transient leaf infiltration-based expression and stable expression in whole plants. By re-designing the plant root architecture (the length and branching pattern), soil anchorage and water and nutrient absorption may be improved, enabling the plants to tolerate drought stress and prevent lodging.

## Conclusion and future perspectives

Plant developmental engineering is an emerging field that requires tight spatiotemporal control over target genes. Recent advances in synthetic biology and the development of tools, such as DNA assembly toolkits, cell- or tissue-type-specific promoters, inducible systems, logic gates, and CRISPR-based multiplex regulation, are paving the way for precise and predictable control over developmental processes. The proof-of-concept studies show that the technology works and is promisingly widely applicable; however, complications are observed in predictive genetic engineering, pointing to certain improvements and further innovations which are needed in the future.

One recurring problem is interactions among parts; biology is full of emergent behaviours, which are recognized even among genetic parts and the host cellular environment. As more standard parts (e.g. promoters and terminators) are made, characterized, and shared in the community, cloning multigene constructs becomes increasingly feasible and accessible. However, a strong promoter was not always found to be strong; its activity changes depending on the paring terminator sequence (Andreou *et al.*, 2021, Preprint). Transgene activity is dependent on chassis type; species to species variations (e.g. monocot versus dicot) are commonly recognized, but even for the same species, a construct behaves differently depending on the cellular context (Brophy *et al.*, 2022; Chamness *et al.*, 2023). Such interactions were also observed intergenically among the different transcriptional units within a construct (Kallam *et al.*, 2023). Together these interactions and context dependency challenge the orthogonality assumption in rational designing of genetic modifications. We recommend validating part activity in each construct, host chassis, and relevant environmental conditions, without assuming the part characterization data in another study will hold generally.

Synthetic promoters and terminators may hold the key to circumventing the interaction problem and enhancing the orthogonality of genetic parts. Currently, the standard parts library is expanding for promoters and terminators, which consist of numerous known and unknown *cis*-elements. If several options of reliably consistent promoters and terminators, deprived of any interactive DNA sequences, can be developed, they will be instrumental for predictive genetic engineering. Another invaluable future resource is a DNA element library—a collection of functional short DNA sequences (e.g. non-promiscuous binding sites for a cell- or tissue type-specific expression). These elements can be used to build developmental logic gates, as in Brophy *et al.* (2022), to induce gene expression in specific cell types, as well as to prevent basal level expression. The recently developed GB_SynP synthetic promoters are rationally designed and can act as synthetic transcription factors in the dCasEV2.1 system (Moreno-Giménez *et al.*, 2022). Inducible systems or logic gates with GB_SynP promoters will be useful in modulating developmental pathways.

Besides the interaction issues, synthetic developmental biology is challenged by transgene instability. Genome-integrated transgenes are often unstable, being genetically rearranged or epigenetically silenced over generations (Finnegan and McElroy, 1994; Dietz-Pfeilstetter, 2010). This problem is not unique to plants and poses a serious problem in multigene modifications common in metabolic or developmental engineering in both plants and animals (Gelvin, 2017; Cabrera *et al.*, 2022). As we deepen our understanding of how the transgenes are recognized to trigger the suppression, we can develop and implement new strategies to reduce such transgene instability. For example, the gene regulatory sequences (promoters, and probably terminators and specific *cis*-elements) seem to affect this, and thus they could be characterized for transgene stability. The genomic integration sites also matter; if a transgene construct lands within or near a heterochromatin region, it is more likely to be silenced. Specific landing pads for ‘high stability’ transgene integration would be beneficial (Danilo *et al.*, 2018; Dhiman *et al.*, 2020). Transgene suppression is intimately linked to defence against pathogens, and its mechanisms are manifold. Some reduction may be possible, but complete suppression of the instability is probably unattainable.

We envisage that plant developmental engineering will contribute to agricultural improvement via two distinct stages. In the first explorative stage, transgene-based pathway engineering is employed to test genetic variations that matter for the output. Synthetic biology tools enable rational engineering of extensive phenotypic variations to survey the parameter space to identify the most effective genetic modifications. Design–build–test–learn cycles will help find the combinations of genetic variants linked to desirable morphology and architecture. In the next stage, gene editing will be used to introduce the identified specific modifications to the genome. Gene editing is highly specific and does not involve transgenes; it is genetically and functionally more stable, and hence better suited to crop and industrial strain improvement. Recently, gene editing has been used successfully to make permanent and highly targeted alterations in plant development, such as the re-creation of multiple key mutations for the domestication of tomato and other crops (Lemmon *et al.*, 2018; Li *et al.*, 2018; Zsögön *et al.*, 2018; Khan *et al.*, 2019). Innovations in synthetic biology technologies to engineer the molecular circuits behind plant development, host chassis biology, and gene editing technology will together bring on future revolutions in agriculture and other forms of plant-based bio-production.

The prime target of plant developmental engineering is root and shoot architecture, as it is thought to directly increase biomass yield and structural stability. Plant body plans may be rationally re-designed through the modulations of developmental master regulators. For instance, the *IDEAL PLANT ARCHITECTURE 1* (*IPA1*) gene has been successfully modified to improve grain yield (Miura *et al.*, 2010), tiller number (Jiao *et al.*, 2010), plant height, and lodging resistance (Lu *et al.*, 2013; Zhang *et al.*, 2017). However, rational engineering of plant development is not straightforward as it may result in counter-productive outcomes. Semi-dwarf statures intended for better structural stability may reduce the number of tillers and grains (Y. Wang e*t al.*, 2015; Jatayev *et al.*, 2020). While one may expect that more branching would lead to more yield, some branches could develop low-quality fruits and seeds, reducing the viable yield (Soyk *et al.*, 2017; Lemmon *et al.*, 2018). In rational engineering of plant development, especially for agronomic traits, structural–functional analysis and optimization of the new morphological features through multiple iterations of design–build–test–learn cycles are anticipated.

Synthetic biology engineering of plant growth and development will probably accelerate the realization of neo-forms better adapted to the changing climate. A number of plants, including clover, pea, and *Oxalis*, develop functional structures to cope with fluctuating environments; they open and close their leaves in response to temperature and light conditions. Engineering crop plants with structures conferring similar leaf closure will help protect them against drought-inducing stressors. In addition to crop improvement, plant developmental engineering may also aid in implementing solutions for environmental problems. The Harnessing Plant Initiative project is one such futuristic project that aims to engineer crop plants to withdraw carbon dioxide from the atmosphere and store it in plant biomass, especially the suberized root tissues (Busch and Miller, 2022). Suberin is thought to be more resistant to microbial degradations than other plant cell wall constituents, and thus is likely to be able to sequester carbon for a longer term. These plants will also be engineered to have increased root biomass and longer roots to absorb more nutrients and water.

With synthetic biology molecular tools and frameworks, it is becoming increasingly possible to realize new biological functions to help solve knotted problems in society and the planet, such as food security and climate crisis. Molecular engineering tools will keep improving, and so will the protocols for transgene introduction and stability, phenotypic analysis, and functional assessment. We are entering an exciting time to re-imagine plant development and learn about it further through iterative investigative cycles.

## Data Availability

No new data were generated or analysed in this paper.
